# Ceragenin-mediated disruption of *Pseudomonas aeruginosa* biofilms

**DOI:** 10.1371/journal.pone.0298112

**Published:** 2024-02-12

**Authors:** Urszula Wnorowska, Dawid Łysik, Ewelina Piktel, Magdalena Zakrzewska, Sławomir Okła, Agata Lesiak, Jakub Spałek, Joanna Mystkowska, Paul B. Savage, Paul Janmey, Krzysztof Fiedoruk, Robert Bucki

**Affiliations:** 1 Department of Medical Microbiology and Nanobiomedical Engineering, Medical University of Białystok, Białystok, Poland; 2 Institute of Biomedical Engineering, Bialystok University of Technology, Bialystok, Poland; 3 Independent Laboratory of Nanomedicine, Medical University of Białystok, Białystok, Poland; 4 Institute of Medical Sciences, Collegium Medicum, Jan Kochanowski University of Kielce, Kielce, Poland; 5 Department of Chemistry and Biochemistry, Brigham Young University, Provo, Utah, United States of America; 6 Department of Physiology and Institute for Medicine and Engineering, University of Pennsylvania, Philadelphia, Pennsylvania, United States of America; University of Pennsylvania, UNITED STATES

## Abstract

**Background:**

Microbial biofilms, as a hallmark of cystic fibrosis (CF) lung disease and other chronic infections, remain a desirable target for antimicrobial therapy. These biopolymer-based viscoelastic structures protect pathogenic organisms from immune responses and antibiotics. Consequently, treatments directed at disrupting biofilms represent a promising strategy for combating biofilm-associated infections. In CF patients, the viscoelasticity of biofilms is determined mainly by their polymicrobial nature and species-specific traits, such as *Pseudomonas aeruginosa* filamentous (Pf) bacteriophages. Therefore, we examined the impact of microbicidal ceragenins (CSAs) supported by mucolytic agents–DNase I and poly-aspartic acid (pASP), on the viability and viscoelasticity of mono- and bispecies biofilms formed by Pf-positive and Pf-negative *P*. *aeruginosa* strains co-cultured with *Staphylococcus aureus* or *Candida albicans*.

**Methods:**

The *in vitro* antimicrobial activity of ceragenins against *P*. *aeruginosa* in mono- and dual-species cultures was assessed by determining minimum inhibitory concentration (MIC) and minimum bactericidal/fungicidal concentration (MBC/MFC). Inhibition of *P*. *aeruginosa* mono- and dual-species biofilms formation by ceragenins alone and in combination with DNase I or poly-aspartic acid (pASP) was estimated by the crystal violet assay. Additionally, the viability of the biofilms was measured by colony-forming unit (CFU) counting. Finally, the biofilms’ viscoelastic properties characterized by shear storage (G’) and loss moduli (G”), were analyzed with a rotational rheometer.

**Results:**

Our results demonstrated that ceragenin CSA-13 inhibits biofilm formation and increases its fluidity regardless of the Pf-profile and species composition; however, the Pf-positive biofilms are characterized by elevated viscosity and elasticity parameters.

**Conclusion:**

Due to its microbicidal and viscoelasticity-modifying properties, CSA-13 displays therapeutic potential in biofilm-associated infections, especially when combined with mucolytic agents.

## Introduction

Biofilm formation is a colonization and survival strategy exploited by numerous microorganisms, such as *Pseudomonas aeruginosa* and other cystic fibrosis (CF) pathogens, often leading to chronic infections. Biophysically, these microbial communities embedded in a matrix of autogenic extracellular polymers (EPS) are characterized by viscoelastic, i.e., either solid (elastic) and liquid (viscous) qualities, that correlate with their resistance to mechanical stress as well as eradication by immune responses and antibiotics [1, 2]. Compared to their planktonic counterparts, bacteria living in biofilms can be up to a thousand times more resistant to antibiotics. Consequently, understanding biofilm viscoelastic properties is crucial for developing effective treatments for biofilm-associated infections.

The remarkable adaptability of biofilms is due to the varied assemblage of species and their EPS matrix components. The latter account for 90% of biofilm biomass, primarily consisting of polysaccharides, extracellular DNA (eDNA) and RNA (eRNA), proteins, and lipids, which collectively are termed the ‘matrix’ [3, 4] Also, host biomolecules, e.g., soluble proteins, glycoproteins, polymeric fibrin, F-actin and DNA, can be incorporated into the matrix, hence its composition and structure adapt to the local environment, availability of nutrients/substrates as well as local shear stress [3, 5]. For instance, highly viscoelastic CF mucus, which is osmotically hyperconcentrated and rich in DNA and mucin, and associated with low mucociliary transport in CF lungs [6, 7], promotes robust biofilm formation by *P*. *aeruginosa* and other CF pathogens, including *S*. *aureus*, *Haemophilus influenzae*, *Burkholderia cepacia* complex (BCC), and *C*. *albicans* [8–11]. The polymicrobial nature of CF biofilms substantially increases matrix heterogeneity, which along with species-specific factors, such as various exopolysaccharides (Psl—*polysaccharide synthesis locus*, Pel-*pellicle polysaccharide*, and alginate) produced by *P*. *aeruginosa*, strongly determine their viscoelasticity [12].

In response, various biopolymer-disrupting mucolytic treatments have been developed, such as the degradation of DNA by DNases and disruption of DNA/F-actin by anionic poly-aspartic acid (pASP) [13–16]. Similarly, targeting *P*. *aeruginosa* filamentous bacteriophages (Pf), carried as prophages by multiple clinical isolates, e.g., Liverpool epidemic strain (LES), may provide an alternative therapeutic approach for CF patients [17]. The presence of Pf phages in sputum was correlated with a higher risk of chronic infection, a more significant decline in pulmonary function during exacerbations, and the development of antibiotic resistance compared to Pf-negative sputum samples [18]. Likewise, Pf-producing *P*. *aeruginosa* strains were associated with delayed healing in patients with chronic wound infections [19], and modulation of immune response toward impairing bacterial clearance at the site of infection [20]. Furthermore, Pf phages exist in two evolutionary lineages (I and II) with significantly different properties, such as the net charge of the capsid protein, which may affect interactions with other matrix biomolecules [21].

As a result, effective treatment of biofilm-associated infections necessitates a combination of agents disrupting structural components of the matrix and killing microbial cells, preferably by antimicrobials that maintain activity in this biomolecule-rich niche. Ceragenins are non-peptide mimics of endogenous antimicrobial peptides (AMPs), developed to overcome the therapeutic limitations of AMPs, e.g., inactivation by anionic biopolymers, high salt concentrations, or proteases [22, 23]. Ceragenins are less susceptible to inactivation by DNA or F-actin compared to AMPs, and their antibacterial action is not impeded by Pf phages [24]. Additionally, ceragenins possess a broad-spectrum microbicidal activity, low resistance potential, and show no cross-resistance to other antimicrobials. Moreover, they act synergistically with conventional antibiotics against multiple bacterial strains, including multidrug-resistant (MDR) ones [25–27], and possess anti-inflammatory properties [28]. Hence, ceragenins can potentially eradicate biofilms in CF airways [28, 29].

To test the ability of ceragenins to disrupt biofilms, we evaluated their antibacterial activity in combination with the viscoelasticity-reducing agents, DNase I and poly-aspartic acid (pASP), against mono- and bi-species biofilms produced by *P*. *aeruginosa* Pf-positive and Pf-negative strains with either *S*. *aureus* or *C*. *albicans*. We show that the treated biofilms are more vulnerable to the microbicidal action of ceragenins, likely due to reduced viscoelasticity, regardless of the species composition and Pf-patterns of *P*. *aeruginosa* strains.

## Materials and methods

### Microbial strains

The study involved clinical strains of *P*. *aeruginosa* (n = 10) isolated from sputum samples collected from CF patients at the Adult Cystic Fibrosis Center at the University of Pennsylvania Health System (recruitment of participants were between 8/5/2005-5/23/2008) in the United States. Sputum samples and bacterial isolates were obtained with the agreement of The University of Pennsylvania’s Institutional Review Board (IRB) (no. 803255) in compliance with the Helsinki Declaration and written informed consent was obtained from each patient.

The *P*. *aeruginosa* strains were selected based on the presence or absence of filamentous (Pf) phages (denoted hereinafter as Pf-positive and Pf-negative strains, respectively). Briefly, five Pf-positive and five Pf-negative *P*. *aeruginosa* isolates were identified based on PCR technique with universal primers for Pf phages (5’-CCTTGGCSAGRTAGTGGTCT-3’, 5’-CTATTGGGAAAGCGTGTGGG-3’), and prescribed further into five groups based on differences in the integrase genes using specific primers: (i) Pf-tRNA-Gly (5’-CTCTGGTATGAACTGCACGG-3’, 5’-TGATGCTTGGTCAGGTACGA-3’), (ii) Pf-tRNA-Met (5’-TGGACGGCTGACTTCTATGA-3’, 5’-TGATCCAGGGACAGAAACGT-3’), (iii) Pf-tRNA-Sec (5’-GTTTCAAGACCAAGGGCGAA-3’, 5’-CAGGCGTTGTTCCAGTTCTG-3’), (iv) Pf-tmRNA (5’-AAGCGTTTCAAGACCAAGGG-3’, 5’-ATTGTTAGCGTCGAATGCCC-3’), (v) Pf-DR (5’-CCCCGTTCAGCAAGATAAGC-3’, 5’-CGGATCGAACACGCTTTGAT-3’) to identify isolates with unique Pf-profiles. The primers were designed based on our previous paper [21], and the PCR reaction was performed with MasterGradient thermocycler (Eppendorf, Germany) and DreamTaq Green PCR Master Mix (Thermofisher Scientific, USA). PCRs were performed in 50 μL volumes containing the following components: 1x DreamTaq Green PCR Master Mix, specific primers at concentration 0.5 μM, and 5 μL of bacterial genomic DNA—isolated from bacterial overnight cultures in LB broth with a Genomic Mini AX Bacteria Spin Kit (A&A Biotechnology, Poland). The PCR cycle conditions for all reactions were as follows: 95°C for 5 min, followed by 35 cycles of 95°C for 30 s, 60°C for 30 s, and 72°C for 60 s, and a final extension at 72°C for 5 min. Amplification was performed on a MasterGradient thermocycler (Eppendorf, Germany). All PCRs were done in triplicate. PCR products were visualized by electrophoresis on a 2% agarose gel stained with SimplySafe stain (EURx, Poland) and documented by GelDoc 2000 system (Bio-Rad, USA). In addition, the phyloproteomic relatedness of the strains was assessed by proteomic profiling using MALDI-TOF MS (Vitek MS, Biomerieux, France) and ‘taxonomy module’ in Saramis v4.12 Vitek MS-Plus RUO software.

The antibiotic susceptibility of *P*. *aeruginosa* isolates was determined using the VITEK^®^2 Compact system and Gram-negative Susceptibility Cards AST-N331 (bioMérieux, Marcy-l’Etoile, France), and interpreted using EUCAST ver. 11 guidelines. *Staphylococcus aureus* Xen30 (Caliper Life Sciences, Hopkinton, MA, USA) and *Candida albicans* 1408 (Polish Collection of Microorganisms, Polish Academy of Science, Wroclaw, Poland) were used to develop dual-species biofilm models.

### Evaluated compounds

Three ceragenins (CSA-13, CSA-44, and CSA-131) were synthesized from cholic acid as previously described [30]. Poly (D, L-aspartic) acid (pASP; molecular weight 2,000–11,000; catalogue no. P3418) and bovine pancreatic DNase I were supplied by Sigma-Aldrich (St. Louis, MO, USA; catalogue no. D5025). Pf1 bacteriophage was from ASLA Biotech (Latvia; catalogue no. P-100). All compounds were stored according to the manufacturer’s instructions.

### Determination of CSAs activity against the planktonic growth of *P*. *aeruginosa* in mono- and dual-species cultures

The minimum inhibitory concentrations (MIC) for CSA-13, CSA-44, and CSA-131 were determined in 96-well microtiter plates using two-fold serial dilutions of the ceragenins, ranging from 0.25 to 256 μg/mL, in Mueller Hinton (MH) and a final *P*. *aeruginosa* density of 5 × 10^5^ cfu/mL. To do so, bacteria at the logarithmic phase of growth were collected and adjusted in LB broth to the OD ~ 0.1. The plates were incubated at 37°C for 24 hours, followed by visual inspection for the MIC values, i.e., concentrations with no observable bacterial growth. In addition, two randomly selected Pf-positive and Pf-negative *P*. *aeruginosa* strains were mixed with *S*. *aureus* X30 or *C*. *albicans* 1408 cells to estimate the antibacterial effects of ceragenins against dual-species cultures using the same procedure.

### Determination of CSAs activity against *P*. *aeruginosa* mono- and dual-species biofilms

To estimate the ability of CSA-13 to suppress *P*. *aeruginosa* biofilm formation, bacterial strains at a final inoculum of 10^5^ CFU/mL were incubated in MH broth with varied doses (5 and 20 μg/mL) of the ceragenins in 96-well microtiter plates to a final volume of 200 μL for 48h at 37°C. After incubation, planktonic cells were removed, and the attached cells were washed three times with PBS. As evidenced in earlier reports, 48-hour incubation in high nutrient medium is sufficient to form a mature biofilm by *Pseudomonas* isolates [31, 32]. The biofilm was stained with 0.1% (w/v) crystal violet. After 15 minutes, the crystal violet solution was removed, and the plates were carefully washed with PBS for 2 seconds. To determine the mass of the biofilm, the remaining biofilm-staining crystal violet was dissolved in 95% ethanol. Biofilm intensity in the 96-well plated were measured at 570 nm using a Labsystem Varioscan Lux (Thermo Fisher Scientific, Waltham, MA, USA). The 50% inhibitory concentrations (IC_50_) were calculated based on percentage inhibition with the different concentrations of CSAs compared with the non-treated biofilm.

In corresponding experiments, the mass of *P*. *aeruginosa* biofilms was evaluated in the presence of DNase I at concentrations ranging from 0.5 to 5 μg/mL, pASP at concentrations ranging from 1 to 200 μg/mL, and Pf1 bacteriophage at concentrations ranging from 0.01 to 0.5 mg/mL, that were administered alone and in combination with CSA-13 at doses of 5 or 20 μg/mL.

Likewise, the same procedure was applied for polymicrobial biofilms formed by Pf-positive and Pf-negative *P*. *aeruginosa* strains co-cultured with *S*. *aureus* and *C*. *albicans*. Recorded values were compared to the anti-biofilm activity of CSA-13 alone.

### Estimation of biofilm viability

A colony-forming units (CFUs) counting technique was used as a complementary method to determine the microbicidal action of the compounds in *P*. *aeruginosa* dual-species biofilms. Briefly, the viability of the biofilms formed by Pf-positive and Pf-negative *P*. *aeruginosa* strains with *S*. *aureus*, and *C*. *albicans* strains was examined in the presence of with CSA-13 at a dose of 20 μg/mL alone and when co-administrated with DNase I, pASP as well as exogenous Pf1 phage at concentrations of 5 μg/mL, 10 μg/mL, and 0.1 mg/mL, respectively. After 48 hours of incubation, biofilms were sonicated for 5 minutes, diluted, and plated on tryptic soy agar plates supplemented with 5% sheep blood. After incubating the plates for at least 24 hours, the number of CFUs per biofilm sample was calculated.

### Evaluation of rheological properties of biofilms

Rheological measurements were performed on a HAAKE Rheostress 6000 rotational rheometer (Thermo Scientific) using a Peltier system to maintain a constant temperature of 21°C. Untreated and treated 10-day-old mono and dual-species biofilms were collected in plastic tubes and applied to the bottom plate of the rheometer. In the measurements of the viscoelastic properties, the forced oscillation method in the plate-plate arrangement (with a plate diameter of 35 mm) was used. In short, the upper plate rotates by a specified angle (shear strain amplitude γ_0_) and frequency (ω), applying oscillatory shear strain to the sample, according to the equation: γ(ω,t) = γ_0_sin(ωt). The stress measured in this way can be described by the equation: τ(ω,t) = G’γ_0_sin(ωt) + G”γ_0_cos(ωt), where G’ is storage shear strain modulus and G” is loss shear strain modulus. The research protocol was as follows: (1) amplitude sweep tests carried out in the range of 0.01–100 with a fixed frequency of 1 Hz, and (2) frequency sweep in the range of 0.1–10 Hz with a fixed amplitude of 0.01 (range of linear viscoelasticity). In addition, viscosity measurements were performed in rotational tests in the cone (diameter 35 mm, angle 1 degree)–plate arrangement, where the dynamic viscosity was determined in the range 0.1–100 1/s of shear rate (linearly related to the rotational cone velocity).

### Statistical analysis

Data are presented and mean ± SE. The significance of differences was determined using the two-tailed Student’s t-test. Statistical analyses were performed using OriginPro 2021 (OriginLab Corporation, Northampton, USA). p <0.05 was considered statistically significant.

## Results

### Filamentous phages (Pf) profiles of *P*. *aeruginosa* strains

All Pf-positive *P*. *aeruginosa* isolates possessed unique Pf-profiles, consisting of one to four Pf phages belonging to four groups according to chromosome integration site, i.e., Pf-tRNA-Gly, Pf-tRNA-Met, Pf-tRNA-DR, Pf-tmRNA. The Pf-profiles and phyloproteomic relatedness of *P*. *aeruginosa* isolates are shown in S1 Fig.

### Ceragenins show high antimicrobial activity against CF-associated *P*. *aeruginosa* strains

**Fig 1:**
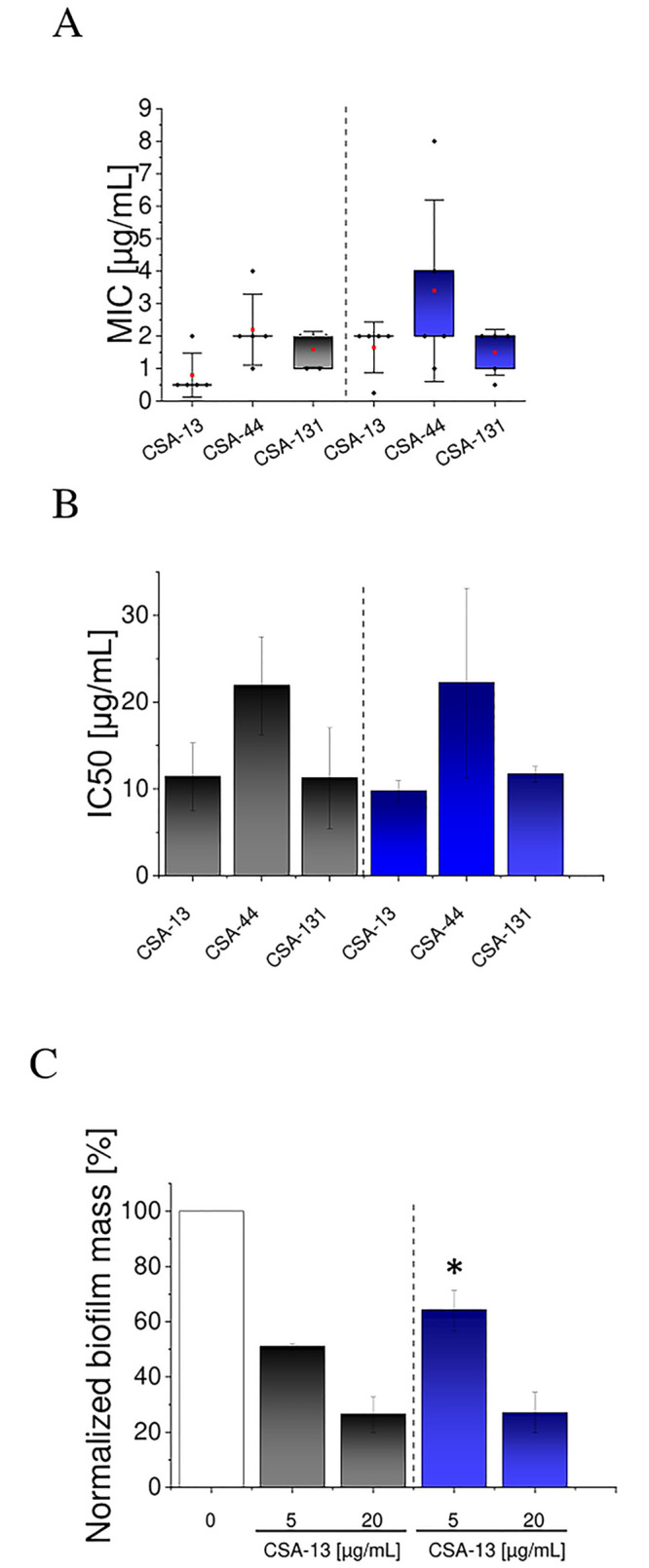
(A) Minimal inhibitory concentrations and minimal bactericidal concentrations (MIC/MBC; μg/mL) of CSA-13, CSA-44, and CSA-131 against five Pf-negative *P*. *aeruginosa* strains (black bars) and five Pf-positive *P*. *aeruginosa* strains (blue bars). (B) Inhibitory concentration at 50%-level (IC50) was recorded for tested ceragenins (CSA-13, CSA-44, and CSA-131) against five Pf-negative *P*. *aeruginosa* strains (black bars) and Pf-positive *P*. *aeruginosa* strains blue bars). The IC50 values were calculated by interpolation of the dose-response curve when biofilm formation in the presence of tested agents was measured. Results are presented as mean ± SD from 5 strains of three repetitions. Results were not statistically significant. (C) Relative mass of biofilm formed by five strains of Pf-negative *P*. *aeruginosa* (black bars) and Pf-positive *P*. *aeruginosa* (blue bars) in the presence of CSA-13 at doses of 5 and 20 μg/mL. Results are presented as mean ± SD from 5 strains of three repetitions. * indicates statistical significance when compared to Pf-negative *P*. *aeruginosa* isolates.

The *P*. *aeruginosa* strains were multidrug-resistant (MDR), defined as strains non-susceptible to at least one agent in three or more antimicrobial categories used for treatment (Table 1) or colistin-only sensitive (COS) pathogens, i.e., resistant to all antipseudomonal agents, except colistin [33, 34]. All three ceragenins, CSA-13, CSA-44, and CSA-131, exhibited potent bactericidal activity, regardless of the antibiotic susceptibility- and Pf-profiles of *P*. *aeruginosa* strains (Fig 1A). Pf-positive *P*. *aeruginosa* isolates were characterized by elevated CSA-13 MIC values (MIC_50_ = 2 μg/mL and MIC_90_ = 2 μg/mL) compared to Pf-negative strains (MIC_50_ = 0.5 μg/mL and MIC_90_ = 2 μg/mL), along with higher MBC/MFC values. However, these differences were not statistically significant.

**Table 1:** The antibiotic susceptibility profiles of *P*. *aeruginosa* strains.

Class of antimicrobials	Antimicrobial agent	*P*. *aeruginosa* Pf-positive/Pf-negative strains		
		Susceptibility category		
		Sensitive	Intermediate	Resistant
Penicilin	Piperacillin	-/-	1/1	4/4
Penicilins / β-lactamase inhibitors	Piperacillin / Tazobactam	1/3	-/-	4/2
Cephalosporins	Cefepime	3/2	-/-	2/3
	Ceftazidime	3/4	-/-	2/1
Carbapenems	Imipenem	2/4	-/-	3/1
	Meropenem	2/3	1/-	2/2
Fluoroquinolones	Ciprofloxacin	2/-	-/-	3/5
	Levofloxacin	2/1	-/-	3/4
Aminoglycosides	Gentamicin	2/2	-/-	3/3
	Tobramycin	4/3	-/-	1/2
	Amikacin	1/2	2/1	2/2
Polymyxins	Colistin	5/5	-/-	-/-

### Ceragenins inhibit the formation of *P*. *aeruginosa* biofilms

As shown in Fig 1B, there was no statistically significant variability between Pf-positive and Pf-negative *P*. *aeruginosa* strains in the 50%-level inhibitory concentration of ceragenins required to inhibit biofilm formation. CSA-13 exerted the most pronounced anti-biofilm action, with 50%-level inhibitory concentration at 11.35 μg/mL ± 3.91 and 9.71 μg/mL ± 1.22 for Pf-positive and Pf-negative *P*. *aeruginosa* groups, respectively. Based on this, ceragenin CSA-13 was selected for further experiments.

CSA-13 at a concentration of 5 μg/mL reduced the biofilm mass of Pf-negative isolates 13% ± 6% more than Pf-positive ones (Fig 1C). However, this effect was not confirmed by CSA-13 at a higher concentration—20 μg/mL, where the difference in biofilm masses was only 0.6% ± 0.9%.

### The anti-biofilm action of CSA-13 is enhanced by DNase I and pASP

**Fig 2:**
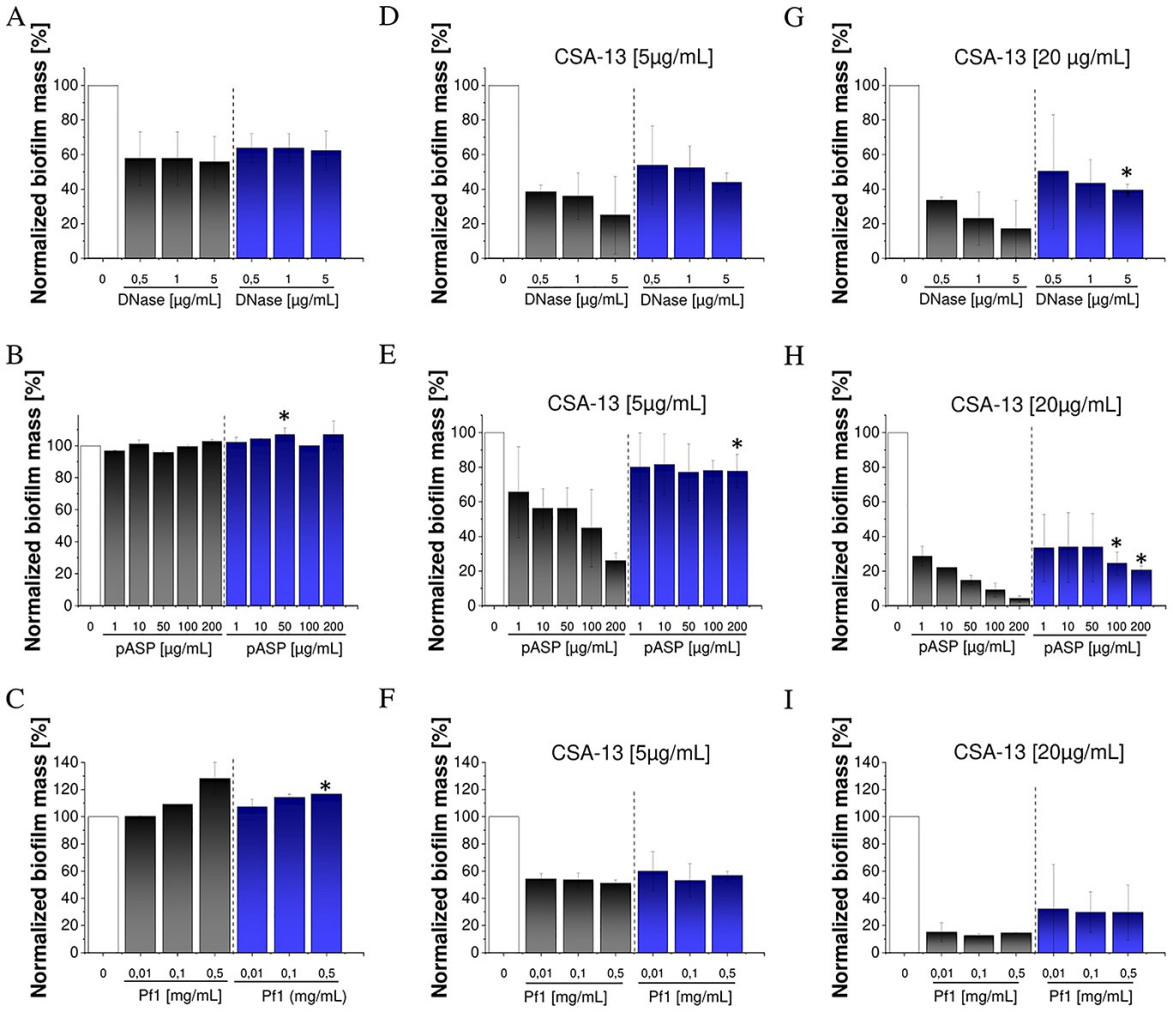
The relative mass of biofilms formed by five strains of Pf-negative *P*. *aeruginosa* (black bars) and Pf-positive *P*. *aeruginosa* (blue bars) in the presence of DNase I at concentrations from 0.5 to 5 μg/mL (A), pASP at concentrations from 1 to 200 μg/mL (B) and Pf1 bacteriophage at concentrations from 0.01 to 0.5 mg/mL (C), both alone and when co-administrated with CSA-13 at doses of 5 (D, E, F) or 20 μg/mL (G, H, I). Results are presented as mean ± SD from 5 strains of three repetitions. *indicates statistical significance when compared to Pf-negative *P*. *aeruginosa* isolates.

DNase I at concentrations of 0.5–5 μg/mL reduced the biofilm mass of Pf-negative and Pf-positive *P*. *aeruginosa* strains by 42–44% and 36–38%, respectively (Fig 2A). In contrast, pASP acid only slightly reduced biofilm formation in the Pf-negative group and appears to even promote biofilm formation in the Pf-positive group (Fig 2B). When a combination of DNase I or pASP acid with CSA-13 was applied, a large decrease in biofilm mass, particularly for CSA-13 at a concentration of 20 μg/mL, was observed. CSA-13 in combination with DNase I at concentrations of 0.5, 1, and 5 μg/mL decreased the biofilm mass of Pf-negative isolates by 66%, 77%, and 82%, and the biofilms of Pf-positive strains were reduced by 50%, 57% and 61%, respectively (Fig 2G). A similar combination of CSA-13 with pASP acid at a concentration of 10 μg/mL reduced the biofilm mass in Pf-negative and Pf-positive *P*. *aeruginosa* groups by 78% and 66%, respectively (Fig 2H). As shown in Fig 1C, administration of the exogenous Pf1 phage resulted in a dose-dependent increase of biofilm mass. However, this biofilm-promoting effect can be diminished by the co-administration of CSA-13 (Fig 2F and 2I).

**Fig 3:**
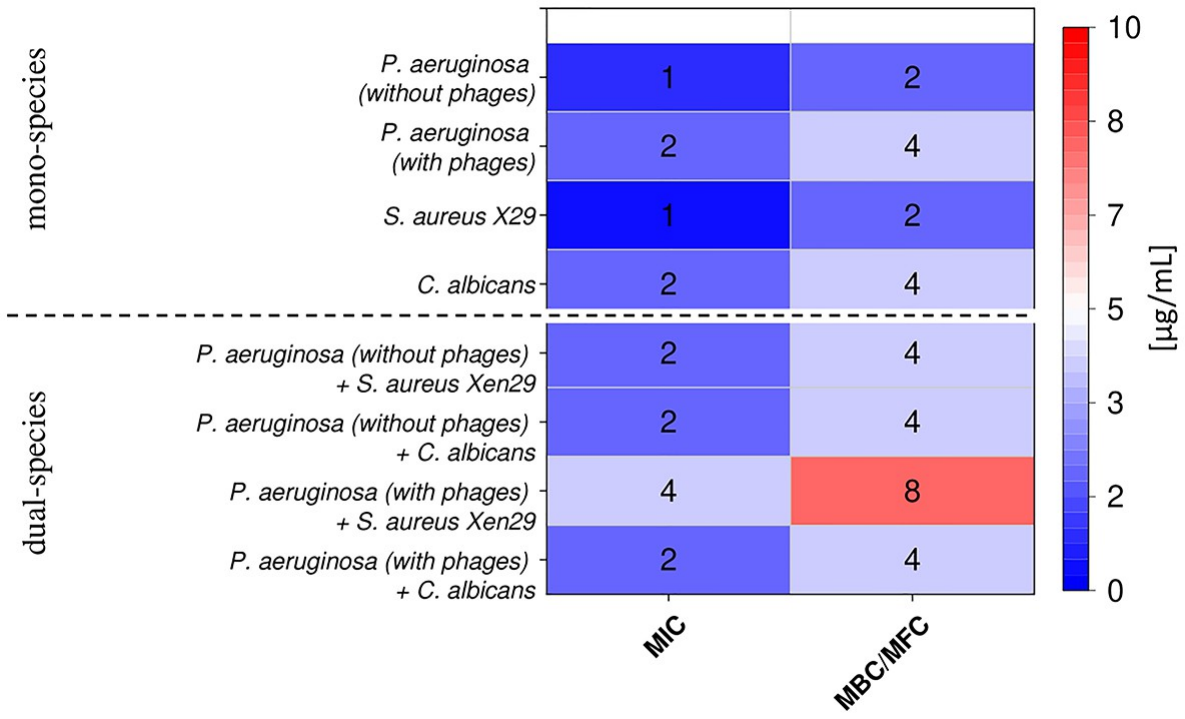
Minimal inhibitory concentration (MIC; μg/mL) and minimal bactericidal/fungicidal concentrations (MBC/MFC; μg/mL) of CSA-13 against Pf-negative *and* Pf-positive *P*. *aeruginosa* isolates, *S*. *aureus* Xen30 and *C*. *albicans* 1408 both in single-species culture or polymicrobial conditions.

**Fig 4:**
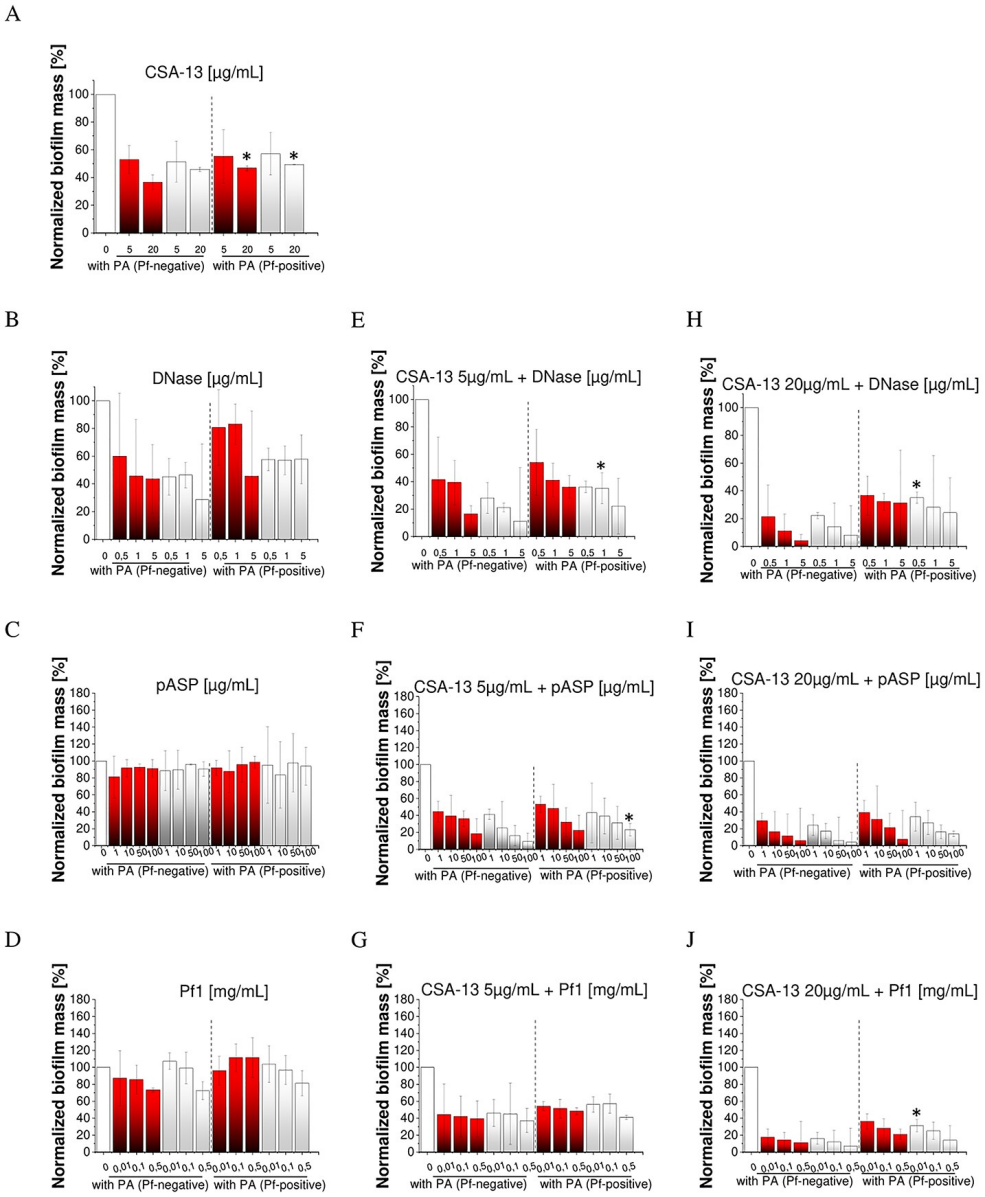
The relative mass of polymicrobial biofilms formed by Pf-negative *and* Pf-positive *P*. *aeruginosa* isolates with *S*. *aureus* Xen30 (SA, red bars), and *C*. *albicans* 1408 (CA, grey bars) strains in dual-species mixtures. Recorded values were compared to the anti-biofilm activity of CSA-13 alone (A). Biofilm mass was measured in the presence of DNase I (B), pASP (C), and Pf1 bacteriophage (D) at concentrations from 0.5 to 5 μg/mL, 1 to 200 μg/mL and 0.01 to 0.5 mg/mL, respectively and co-administrated with CSA-13 at doses of 5 (E-G) or 20 μg/mL (H-J). Results are presented as mean ± SD of three repetitions. * indicates statistical significance when compared to dual-species mixtures of biofilm with Pf-negative *P*. *aeruginosa* isolates.

For dual-species biofilms with *S*. *aureus* and *C*. *albicans*, two strains of *P*. *aeruginosa*, i.e., one Pf-positive and one Pf-negative, were randomly selected. As shown in Fig 3, the MIC/MBC(MFC) values for dual-species, co-cultures, increased insignificantly by one dilution but did not exceed 8 μg/mL, i.e., they remained in the non-toxic concentration range. Inhibition of dual-species biofilm formation by CSA-13 alone and combined with DNase I and pASP acid as well as in biofilms supplemented with exogenous Pf1 phage was recorded (Fig 4A–4J). In general, the activity of the combination of CSA-13 with DNase I and pASP acid on dual-biofilms was similar to that exerted on the single-species biofilms, and considerable differences between biofilms formed by Pf-positive and Pf-negative isolates were noted only for selected concentrations of these compounds. For instance, CSA-13 only at a concentration of 20 μg/mL significantly reduced the mass of *P*. *aeruginosa* Pf-negative dual-biofilms formed with *S*. *aureus* and *C*. *albicans* strains (Fig 4A and S2A Fig). Furthermore, CSA-13, in combination with DNAse I or pASP acid, significantly decreased only the biofilm mass produced by Pf-negative *P*. *aeruginosa* with *C*. *albicans* (Fig 4E, 4F, 4H and 4J, and S2B and S2C Fig). CSA-13 activity was retained in dual-biofilms supplemented with the exogenous Pf1 (Fig 4G–4J and S2D Fig).

**Fig 5:**
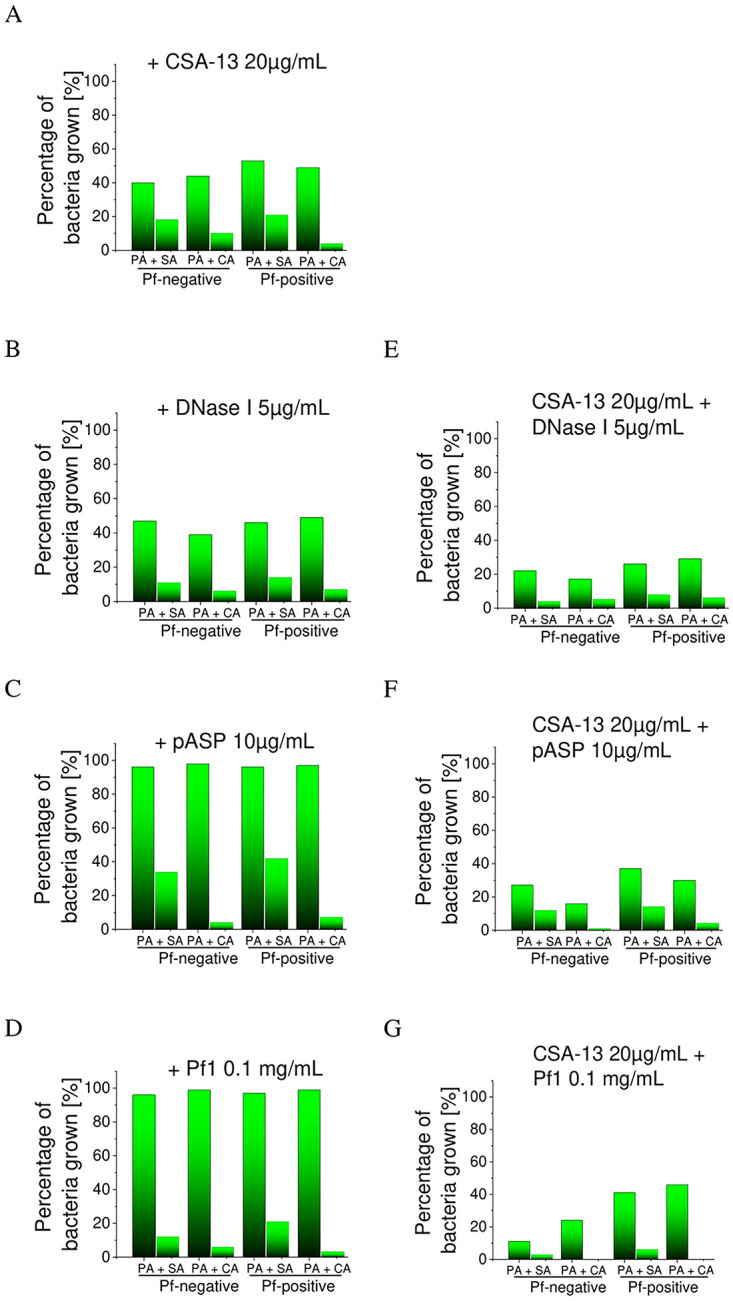
Viability of polymicrobial biofilms formed by Pf-negative *and* Pf-positive *P*. *aeruginosa* isolates, *S*. *aureus* (SA), and *C*. *albicans* (CA) strains in dual-species mixtures. Recorded values were compared to the anti-biofilm activity of CSA-13 alone (A). Biofilm viability was measured in the presence of DNase I (B), pASP (C) and Pf1 bacteriophage (D) at concentrations of 5 μg/mL, 10 μg/mL, and 0.1 mg/mL, respectively and co-administrated with CSA-13 at dose of 20 μg/mL (E-G). Green bars indicate percentage of bacteria grown. Results are presented as mean ± SD of three repetitions.

Viability of mono- and dual-species biofilms, assessed by CFU counting, (Fig 5A–5G and S3A–S3G Fig) revealed that DNase I (Fig 5B and S3B Fig) and pASP (Fig 5C and S3C Fig) are more effective against *S*. *aureus* and *C*. *albicans* than against *P*. *aeruginosa*.

### Viscoelasticity of biofilms depends on *P*. *aeruginosa* Pf-profile

**Fig 6:**
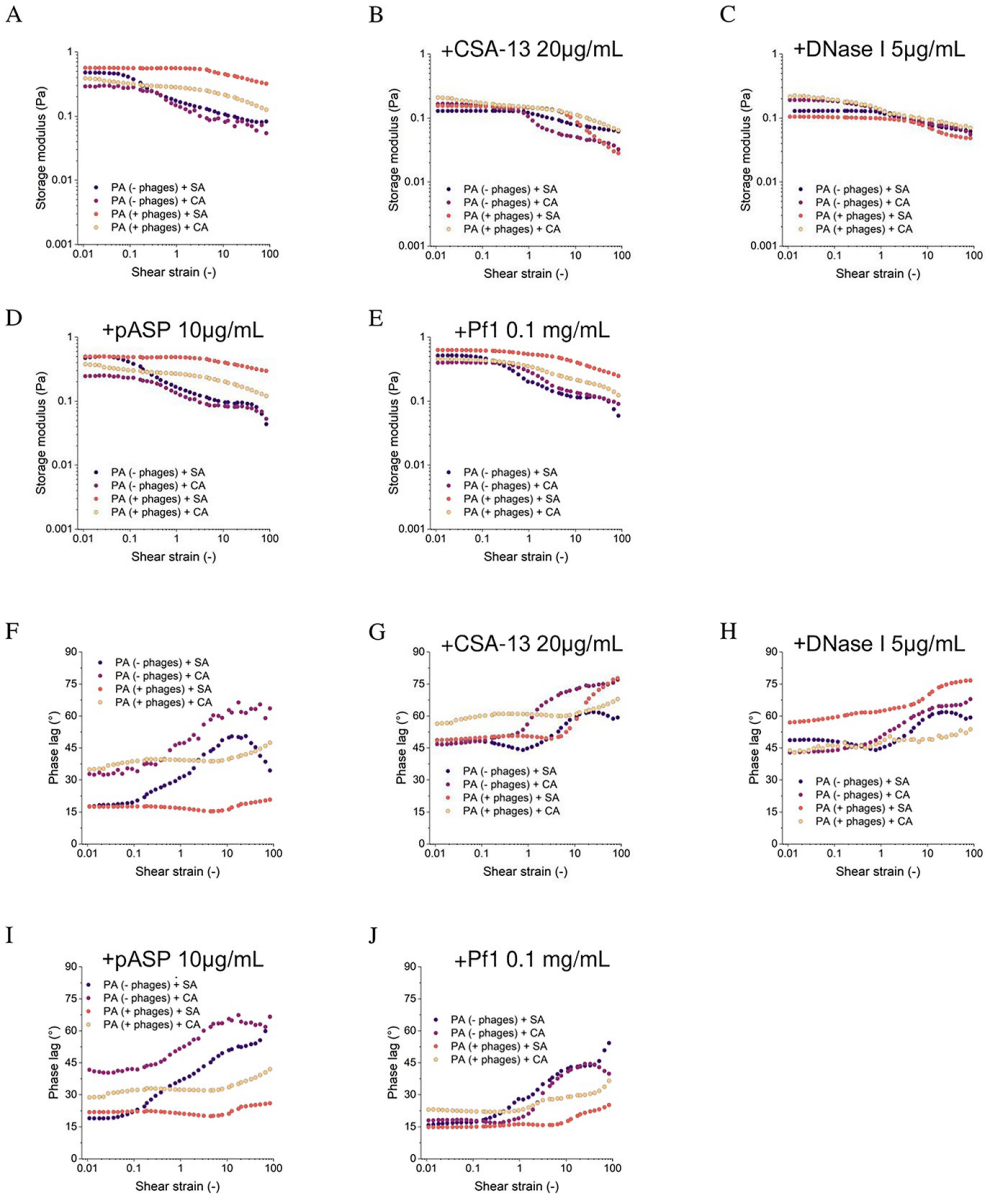
Rheological measurements of polymicrobial biofilms formed by Pf-negative and Pf-positive *P*. *aeruginosa* isolates with *S*. *aureus*, and *C*. *albicans* strains in dual-species mixtures upon treatment with single compound. Shear storage modulus as a function of shear strain amplitude (A-E) and the phase shift as a function of shear strain amplitude (F-J). Rheological properties of biofilms were measured in the presence of CSA-13 (B, G), DNase I (C, H) pASP (D, I) and Pf1 bacteriophage (E, J) at concentrations of 20 μg/mL, 5 μg/mL, 10 μg/mL and 0.1 mg/mL, respectively. Recorded values were compared to the control—without treatment (A, F).

Untreated mono- and dual-biofilms developed by Pf-positive and Pf-negative *P*. *aeruginosa* co-cultured with *S*. *aureus*/*C*. *albicans* strains differ in rheological properties, as shown in S4A–S4D Fig as well as Fig 6A and 6F, respectively. The co-culture of Pf-positive *P*. *aeruginosa* and *S*. *aureus* or *C*. *albicans* results in biofilms with significantly higher rigidity compared to co-cultures with Pf-negative *P*. *aeruginosa* (Fig 6A). Pf-positive *P*. *aeruginosa* biofilms, especially those co-developed with *S*. *aureus*, have a higher storage modulus and a smaller decrease in the amplitude sweep test. For Pf-negative *P*. *aeruginosa* + *S*. *aureus*, G’ values decreases from 0.47 Pa to 0.08 Pa, for Pf-negative *P*. *aeruginosa* + C. albicans G’ values decreases from 0.29 Pa to 0.05 Pa, for Pf-positive *P*. *aeruginosa* + *S*. *aureus* G’ values decreases from 0.56 Pa to 0.32 Pa, and for Pf-positive *P*. *aeruginosa* + *C*. *albicans* G’ values decreases from 0.38 Pa to 0.12 Pa (Fig 6A). Biofilms of Pf-negative isolates show an increase in phase shift above strain 0.1 from ~15° to over 45° at strain 10 for Pf-negative + *S*. *aureus* (followed by a decrease to 30° at strain 100) and from 30° to over 60° for Pf-negative *P*. *aeruginosa* + *C*. *albicans*. A gentle increase from 17° to 21° (Pf-positive *P*. *aeruginosa* + *S*. *aureus*) and from 34° to 47° (Pf-positive *P*. *aeruginosa* + *C*. *albicans*) was observed for biofilms with phages (Fig 6F). The curves of storage modulus as a function of frequency for untreated biofilms are similar, and a linear (on a logarithmic scale) increase in storage modulus from 0.06–0.08 Pa at 0.1 Hz to 15.4 Pa for Pf-negative *P*. *aeruginosa* + *S*. *aureus*, 9.8 Pa for Pf-negative *P*. *aeruginosa* + *C*. *albicans*, 21 Pa for PA (+) + SA and 17.5 for Pf-negative *P*. *aeruginosa* + CA at 10 Hz was not observed (S5A Fig). These observations correspond to the viscosity results, where higher viscosity was observed for PA biofilms with phages (0.38–0.10 Pa or Pf-positive *P*. *aeruginosa* + *S*. *aureus*, 0.23–0.08 Pa for Pf-positive *P*. *aeruginosa* + *C*. *albicans*) than Pf-negative *P*. *aeruginosa* (0.24–0.05 Pa for Pf-negative *P*. *aeruginosa* + *S*. *aureus*, 0.16–0.06 Pa for Pf-negative *P*. *aeruginosa* + *C*. *albicans*) over the entire range of shear rates tested (S5F Fig).

### CSA-13 and DNase I decrease the stiffness and viscosity of biofilms

In the shear strain range of 0.01–1, a 4-fold decrease in storage modulus was observed for Pf-negative *P*. *aeruginosa* + *S*. *aureus* and nearly 2-fold for the other types (Fig 6B). The phase shift over the entire range increased above 45° for all samples, indicating a transition of the biofilm to a more fluid-like consistency (Fig 6G). Similar observations were yielded by frequency sweep tests, wherein the range of 0.1–10 Hz the storage modulus values decreased more than 4 times for Pf-negative *P*. *aeruginosa* + *S*. *aureus*, more than twice for Pf-negative *P*. *aeruginosa* + *C*. *albicans*, and Pf-positive *P*. *aeruginosa* + *C*. *albicans*, and nearly 3 times for Pf-positive *P*. *aeruginosa* + *S*. *aureus* (S5B Fig). The viscosity of CSA-treated biofilms also decreased, especially evident for Pf-positive *P*. *aeruginosa* + *S*. *aureus* and Pf-positive *P*. *aeruginosa* + *C*. *albicans* where a twofold decrease in viscosity was observed in the range up to 1 1/s. For Pf-negative *P*. *aeruginosa* + *S*. *aureus* and Pf-negative *P*. *aeruginosa* + *C*. *albicans*, the decrease in viscosity in this range was 2 and 1.5 times, respectively. At higher shear rates, viscosity was similar to untreated biofilms (S5G Fig). The addition of DNase I also reduces viscosity, especially for biofilms with *S*. *aureus*, for which an almost 4-fold and 5-fold decrease in the storage modulus for Pf-negative and Pf-positive *P*. *aeruginosa* isolates, respectively (Fig 6C) was observed. For *P*. *aeruginosa* with *C*. *albicans*, the storage modulus decreased by about 30% for Pf-negative *P*. *aeruginosa* and 45% with Pf-positive isolates (Fig 6C). Biofilm viscosity also decreased, for Pf-negative *P*. *aeruginosa* + *S*. *aureus* it decreased by almost 70%, for Pf-negative *P*. *aeruginosa* + *C*. *albicans* it decreased by about 30%, for Pf-positive *P*. *aeruginosa* + *S*. *aureus* by over 70%, and for Pf-positive *P*. *aeruginosa* + *C*. *albicans* by nearly 50% (S5H Fig). The addition of pASP slightly affected the rheological behavior of the biofilm. The only significant differences were observed in the overall decrease of several percent in the phase shift of Pf-positive *P*. *aeruginosa* + *C*. *albicans* and the more viscous behavior of Pf-negative *P*. *aeruginosa* + *S*. *aureus* in the range above 10 shear strain (Fig 6D, S5I Fig). The addition of Pf1 had a greater impact on the biofilm mechanics, increasing the storage modulus by several percent and significantly shifting the phase lag of *C*. *albicans* biofilms (by about 15° for Pf-negative *P*. *aeruginosa* + *C*. *albicans* and by about 11° for Pf-positive *P*. *aeruginosa* + *C*. *albicans*) towards more elastic behavior.

### Ceragenin CSA-13 in combination with DNase I and pASP alters mechanical properties by fluidizing mono- and dual-species biofilms

**Fig 7:**
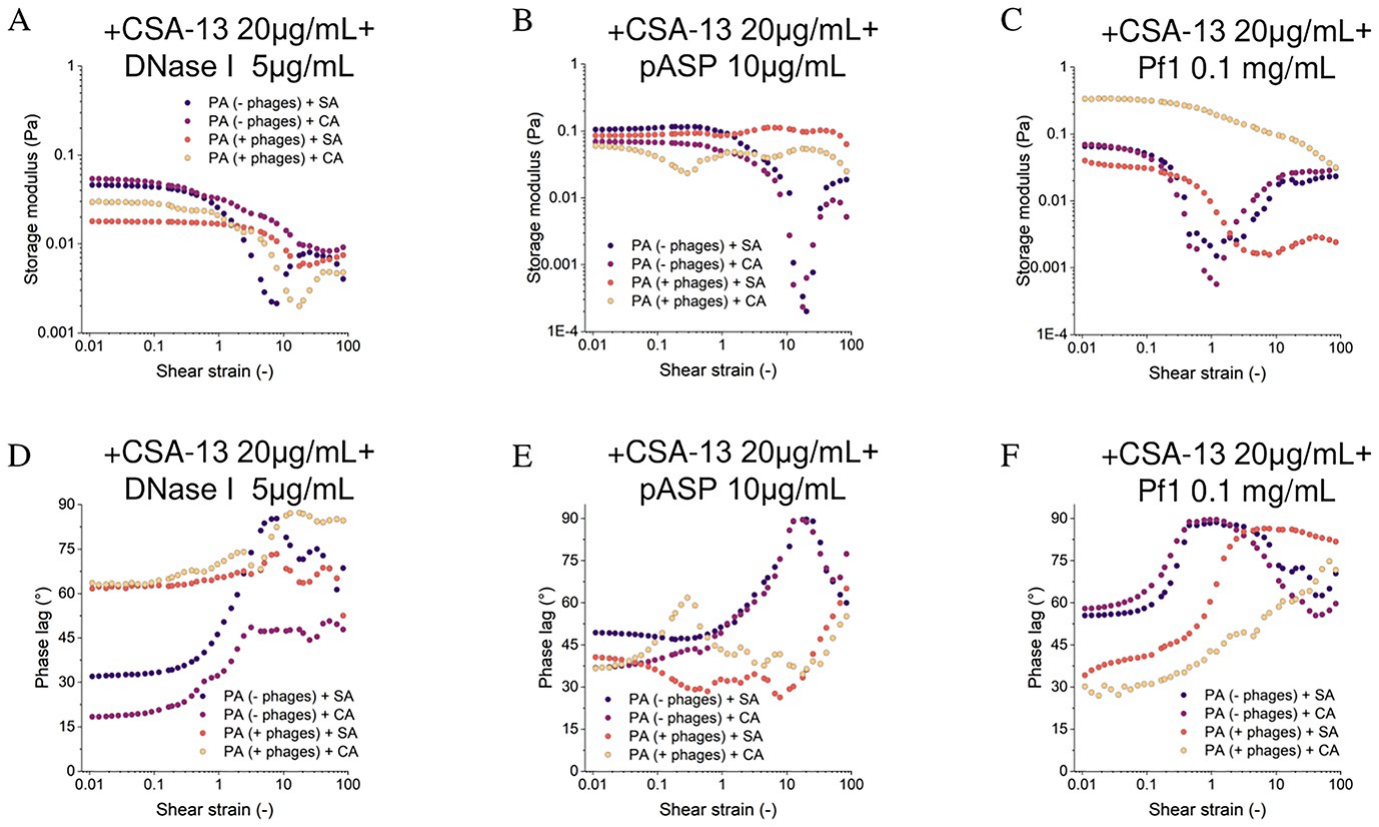
Rheological measurements of polymicrobial biofilms formed by Pf-negative *and* Pf-positive *P*. *aeruginosa* isolates with *S*. *aureus*, and *C*. *albicans* strains in dual-species mixtures upon treatment with co-administrated agents. Shear storage modulus as a function of shear strain amplitude (A-C) and the phase shift as a function of shear strain amplitude (D-F). Rheological properties of biofilms were measured with CSA-13 at dose of 20 μg/mL in the co-administrated with DNase I (A, D) pASP (B, E) and Pf1 bacteriophage (C, F) at concentrations of 5 μg/mL, 10 μg/mL and 0.1 mg/mL, respectively.

Fig 7 shows the rheological behavior of biofilms of the same strain configurations co-treated with CSA-13 and DNase I, pASP, or Pf1. The addition of CSA-13 and DNase I significantly reduced the storage modulus of biofilms for Pf-negative *P*. *aeruginosa* + *S*. *aureus* by over 90%, for Pf-negative *P*. *aeruginosa* + *C*. *albicans* by over 80%, for Pf-positive *P*. *aeruginosa* + *S*. *aureus* by over 95% and for Pf-positive *P*. *aeruginosa* + *C*. *albicans* by over 90% (Fig 7A). At the same time, for all types of biofilms, a sharp decrease in the storage modulus in the shear deformation range of >1 was observed. The change in rheology is also visible in the phase lag values, for Pf-positive *P*. *aeruginosa* it increased above 60° in the range <0.1 of tangential strain, while for Pf-positive *P*. *aeruginosa* + *C*. *albicans* values close to completely viscous liquid were observed (Fig 7D). For Pf-negative *P*. *aeruginosa*, the phase lag did not change significantly in the range <0.1, while >1 a significant increase was observed, especially for Pf-positive *P*. *aeruginosa* + *S*. *aureus* to a value close to 90° at ~10 shear strain. The viscosity of the biofilms decreased over the entire range from below 0.005 Pa for low shear rates to below 0.002 Pa for high shear rates (S6D Fig).

Although pASP alone did not change the rheological behavior of the biofilm, it had a greater effect in combination with CSA-13 than CSA-13 alone (Fig 7B and 7E; S6B and S6E Fig). This combination reduced the storage modulus of biofilms (in terms of small <0.1 deformations in the amplitude sweep and frequency sweep), for Pf-negative *P*. *aeruginosa* + *S*. *aureus* by nearly 80%, for Pf-negative *P*. *aeruginosa* + *C*. *albicans* by over 75%, for Pf-positive *P*. *aeruginosa* + *S*. *aureus* and for Pf-positive *P*. *aeruginosa* + *C*. *albicans* by nearly 85%. For biofilms without bacteriophages, a rapid decrease in storage modulus was observed, accompanied by a rapid increase in phase lag in the range of >1 shear strain. The viscosity of these biofilms fell below 0.01 Pa over the entire range of shear rates (S6E Fig).

Significant rheological changes were also observed after treatment of biofilms with CSA-13 + Pf1 and are greater than when CSA-13 alone was used. In the range of small strains in the amplitude sweep and frequency sweep tests, a decrease in the storage modulus below 0.1 Pa was observed for all tested biofilms except for Pf-positive *P*. *aeruginosa* + *C*. *albicans*, for which the values were close to the control (untreated) (Fig 7C and 7F). For these biofilms, a sharp decrease in storage modulus and an accompanying increase in phase lag >0.1 tangent strain was also observed. The Pf-positive *P*. *aeruginosa* + *C*. *albicans* biofilm viscosity did not decrease significantly below the values obtained for the control, while for the remaining biofilms a decrease below 0.1 Pa was observed in the entire range of tangential deformations (S6E Fig).

## Discussion

Mixed biofilm-associated infections, such as those observed in cystic fibrosis (CF) lung disease, require broad-spectrum and microbicidal antimicrobials that retain activity in polymer-rich environment [35–38]. The viscoelasticity of CF biofilms is strongly influenced by species-specific factors, such as various *P*. *aeruginosa* exopolysaccharides (Psl, Pel, and alginate) and filamentous (Pf) bacteriophages [12, 17]. For instance, Psl promotes the growth of elastic biofilms with highly efficient cross-linking, whereas Pel and alginate favor the formation of viscoelastic and loose biofilms [12, 39, 40]. Pel and Psl polymers also differently mediate *P*. *aeruginosa* interactions in mixed-species biofilm models, e.g., with *S*. *aureus* [39] which, along with species variable susceptibility patterns, promote the development of high tolerance to antimicrobial agents [41, 42], and significantly impede the treatment of CF patients [35–37]. Similarly, Pf bacteriophages elevate biofilm viscosity and create protective nanochambers enclosing bacterial cells [18, 43]. In addition, these ~2 μm long wire-shaped viruses interact with various biofilm polymers (DNA, actin, mucin, and glycosaminoglycans), resulting in a highly ordered liquid crystal formation promoting bacterial adhesion, desiccation survival, and antibiotic tolerance [44]. The latter appears to be mediated via (i) low antibiotic penetration through lattice-like networks as a diffusion-limiting barrier, (ii) bacterial cell encapsulation in liquid crystalline droplets (nanochambers), and (iii) sequestration of cationic antibiotics, like aminoglycosides, by negatively charged virions [18, 43, 44]. Recently, it was discovered that *P*. *aeruginosa* Pf1 and Pf4 phages act as an iron (Fe) chelators and inhibit *C*. *albicans* and *Aspergillus fumigatus* metabolism and biofilm formation via iron sequestration and denying this essential micronutrient to the fungi [45, 46]. This finding has potential practical implications since *C*. *albicans* is a prominent fungal pathogen in CF airways [8–11, 47]. Therefore, anti-biofilm therapies commonly involve various adjuvants that facilitate the action of antimicrobial agents, either by releasing the microbial cells from the protective matrix or by preventing their interactions with its components. Furthermore, novel antibacterial and antifungal compounds, such as essential oils derived from aromatic and medicinal plants, are continuously evaluated as a valuable natural alternative to conventional antibiotics [48–51].

Consistently, we evaluated the efficacy of the bactericidal and fungicidal ceragenin CSA-13 in combination with mucolytic agents–DNase I and pASP acid, against *P*. *aeruginosa* mono- and bi-species (*S*. *aureus* and *C*. *albicans*) biofilms. To address the significance of *P*. *aeruginosa* filamentous (Pf) bacteriophages–a strain-specific biofilm viscoelastic trait—biofilms formed by Pf-positive and Pf-negative *P*. *aeruginosa* isolates were characterized by rheology and compared with the biofilms artificially supplemented with the exogenous Pf1 bacteriophage.

In general, Pf-positive *P*. *aeruginosa* mono- and dual-species biofilms had elevated viscosity and elasticity parameters when compared to Pf-negative ones (Figs 6A, 6E, 6J, 7C and 7F; S5E, S5J, S6C and S6F Figs). Pf-positive biofilm masses were less affected by CSA-13, either alone or in combination with DNase I, pASP acid, and exogenous bacteriophage Pf1. However, statistical significance was observed only for some concentrations of these compounds.

For instance, CSA-13 at a concentration of 5 μg/mL significantly decreased biofilm mass in the Pf-negative *P*. *aeruginosa* group but not at four times higher concentration (20 μg/mL). The total saturation of the biofilm matrix by this amount of CSA-13, and a massive reduction in biofilm mass in both groups, as with conventional antibiotics, is a plausible explanation for this observation [52]. On the other hand, in mixed-species *P*. *aeruginosa*, *S*. *aureus*, and *C*. *albicans* biofilms, a significant impact of Pf phages was recorded only for CSA-13 at a dose of 20 μg/mL. This result might be related to the enhanced resistance of multi-species *P*. *aeruginosa* biofilms to conventional antibiotics, such as flucloxacillin, ciprofloxacin, and fluconazole, mediated by diffusion-limiting effects and/or the presence of matrix-associated degradative enzyme [3]. DNase I improved the efficiency of CSA-13 at concentrations of 5 and 20 μg/mL but these differences were not statistically significant in Pf-positive group.

In contrast, the highest doses of both compounds significantly affected the biofilms of Pf-negative isolates (Fig 2H). This result might be related to enhanced biofilm penetration by CSA-13 due to eDNA degradation and, as a result, lower acidity of the matrix, as with other antimicrobial agents [2]. At the same time, Pf phages can shield bacterial cells by enclosing them in nanochambers made of liquid crystalline droplets [43, 44]. The nanochambers are an example of a higher-order structure formed by filamentous phages due to their spontaneous self-assembly due to crowding or depletion-mediated attraction mechanisms, which are facilitated by a wide range of biopolymers (eDNA, alginate, or hyaluronan), and significantly impacted by the shape and size complementarity of the liquid crystals with rod-shaped bacterial cells [43, 44]. The range and intensity of depletion attraction are proportional to the size and concentration of biopolymers, thus, larger and/or more concentrated polymers favor liquid crystal assembly from lower Pf phage concentrations. Therefore, the degradation of eDNA by DNase I might introduce quantitative and qualitative alterations in the composition of biopolymers that contribute to the formation of liquid crystal nanochambers, e.g., via the altered ratio of DNA length or between DNA and other biopolymers [53] as well as by reducing cell-cell adhesion [43, 54], leading to increased bacterial survival.

The DNA concentration, the microbial cell morphology, and the number of microorganisms are essential determinants of biofilm mechanics [55, 56]. For instance, compression-stiffening and shear-softening features of biofilms can be recapitulated and modulated by modifying the DNA and microbial cell composition [56]. A higher eDNA-to-cells ratio strengthens the biofilm’s structure and enhances its viscoelastic moduli [56]. Moreover, the level of compression-strengthening varies among microbial cell types and is more noticeable for *P*. *aeruginosa* than for *S*. *aureus* or *C*. *albicans* [56]. Therefore, the presence of various cells in mixed biofilms may interfere with the mechanics of DNA, possibly by providing additional cross-links and restricting the space into which DNA can relocate throughout macroscopic deformation [56]. Likewise, the stiffness of *P*. *aeruginosa* biofilms increases with the concentration of Pf1 bacteriophage [56]. As a result, the viscoelastic properties of biofilms may involve mechanisms other than the interaction of cellular elements with the EPS matrix, as evidenced by the mechanical effects of cross-linking in the presence of bivalent ions and the interaction of biofilm components with Pf bacteriophages [56].

For instance, eDNA forms a filamentous network with a honeycomb-like structure and uniform thickness between bacterial cells [57], which may impair the assembly of Pf phages. eDNA is fundamental for the biofilm interactions with other biopolymers, including the major *P*. *aeruginosa* exopolysaccharides–a neutral Psl and cationic Pel, [58]. For example, eDNA-Psl fibers act as a skeleton supporting bacterial adherence and growth and protecting biofilms against degradation by DNase I [53]. However, Psl molecules can also be cross-linked by the protein CdrA, reinforcing the elastic network of the biofilm matrix [40]. Likewise, Pel stabilizes the matrix by ionic interactions that bind eDNA with the biofilm [59]. Furthermore, eDNA promotes the formation of amyloid fibers in bacterial biofilms, i.e., long and thin structures extending from the surface of the bacterial outer membrane, that sequester small metabolites, including essential for bacterial intercellular communication and biofilm formation quorum-sensing molecules [60].

In contrast, pASP, which like Pf1 is an anionic polyelectrolyte, alone at all concentrations (1–200 μg/mL) has negligible anti-pseudomonal biofilm activity or appears to slightly promote biofilm formation, as reflected by generally minor rheological differences compared to untreated biofilms. Therefore, in terms of biofilm mass, the effect of pASP on *P*. *aeruginosa* biofilm formation is comparable to that mediated by exogenous bacteriophage Pf1 (Fig 2B and 2C). The above is reflected in our previous results, which clearly indicate that the activity of pASP depends on its polycationic charge [61].

Subsequently, the combination of CSA-13 at the dose of 20 μg/mL, even with the highest amounts of pASP acid, i.e., 100 and 200 μg/mL, was not superior over CSA-13 alone in the reduction of biofilm mass in Pf-positive *P*. *aeruginosa* isolates. In contrast, biofilms of Pf-negative *P*. *aeruginosa* strains were significantly more affected by both combinations. It remains to be elucidated whether this difference is related to the depletion attraction phenomenon [44], where more concentrated pASP favors the assembly of Pf phages into liquid crystals. Alternatively, since specific D- and L-amino acid isoforms, including aspartic acid, have anti-biofilm activity [38, 62, 63], it might be assumed that its release via degradation polyaspartic acid may inhibit biofilm formation in Pf-negative *P*. *aeruginosa* isolates.

Since pASP acid is an anionic polyelectrolytes, it might exhibit the same behavior as natural biopolymers present in *P*. *aeruginosa* biofilms, e.g., interacting via acidic (carboxyl) groups with cationic biopolymers [58], and due to the high water absorbency, pASP may promote the formation of highly hydrated biofilms. Furthermore, anionic polyelectrolytes undergo chain conformation changes from globular to a coil in the presence of electrolytes, which compensate for the repulsive Coulomb forces between anionic carboxyl groups responsible for the elongation of polymer chains, resulting at higher ion concentrations in an extensive agglomeration of the polymer and growing particle size [58].

The cationic CSA-13 might interfere with this process in a similar way to the cationic histone protein H2A disturbs interactions between alginate and calcium, leading to increased deformability of an alginate-calcium gel and reflected by decreased storage modulus G’ and loss modulus G”, but increased stress under strain [58, 64]. Indeed, a rapid decrease in storage modulus and increase in phase lag was observed in Pf-negative *P*. *aeruginosa* biofilms treated with the CSA-13/ pASP acid combination (Fig 7B and 7E; S6B and S6E Fig). Like DNase I, ASP oligomers can decrease the elasticity of CF sputum or neutrophil-induced *P*. *aeruginosa* biofilms; however, acting via dissolving actin and DNA bundles by sequestration of cationic histone linking electrostatically these negatively charged biopolymers [5, 16].

Our earlier research demonstrated a substantial reduction of the bactericidal action of natural peptide LL-37 in the presence of exogenous bacteriophage Pf1 as well as by Pf-positive *P*. *aeruginosa* LES strain without impairing ceragenins’ activity [24]. Here we extend this observation over additional *P*. *aeruginosa* isolates expressing Pf belonging to distinct evolutionary lineages and characterized by different structural/morphogenesis properties [21]. The presence of Pf phages promotes biofilm formation (Fig 2C) and alters biofilm rheological properties toward enhanced viscosity and elasticity (Figs 6E, 6J, 7C and 7F; S5E, S5J, S6C and S6F Figs). Therefore, targeting *P*. *aeruginosa* Pf phages might provide an alternative therapeutic approach for CF patients. Moreover, according to our best knowledge, this is the first study comparing the activity of CSA-13 in combination with DNase I and pASP in treating CF-associated bispecies biofilms.

In conclusion, this study confirms and expands the findings of our previous studies on the impact of polyelectrolytes, such as DNA and *P*. *aeruginosa* filamentous (Pf) bacteriophages, on rheological properties of biofilms through the investigation of antibiofilm activity of ceragenins (CSAs) and mucolytic agents–DNase I and pASP [56, 65]. CSA-13 is a potent microbicidal and biofilm fluidizing agent, especially when supported by DNase I or pASP acid, and appears to be a good candidate for further studies in the treatment of biofilms produced by *P*. *aeruginosa* strains, regardless of their Pf-profiles, and other CF-associated bacterial and fungal pathogens. Nevertheless, for a more reliable assessment of ceragenin antibiofilm efficacy, experiments must be performed under conditions mimicking CF lungs, such as artificial sputum media (ASM), synthetic cystic fibrosis media (SCFM), or CF sputum samples. Accordingly, we recently reported that ceragenins preserve bactericidal activity in the environment mimicking CF sputum irrespective of its ionic strength [66]. Furthermore, the effect of the microbial cell morphology [56] and Pf bacteriophages representing various phylogenetic lineages [21] on the viscoelastic properties of biofilms should be addressed in further studies.

## Supporting information

S1 Fig Phyloproteomic relatedness of Pf-negative (blue color) and Pf-positive (green color) *P*. *aeruginosa* strains estimated based on MALDI-TOF mass spectra fingerprints. Analysis was performed using ‘taxonomy module’ in Saramis v4.12 Vitek MS-Plus RUO software.
(TIF)

S2 Fig Relative mass of polymicrobial biofilms formed by Pf-negative *and* Pf-positive *P*. *aeruginosa* isolates (black and blue bars, respectively), *S*. *aureus* (SA, red bars), and *C*. *albicans* (CA, grey bars) strains in dual-species mixtures treated with CSA-13 (A), DNase I (B), pASP (C) and Pf1 bacteriophage (D) at concentrations ranging from 5 to 20 μg/mL, 0.05 to 5 μg/mL, 1 to 100 μg/mL and 0.01 to 0.5 mg/mL, respectively. Results are presented as mean ± SD from 5 strains with three repetitions. * indicates statistical significance when compared to Pf-negative *P*. *aeruginosa* isolates.
(TIF)

S3 Fig Viability of biofilms formed by *P*. *aeruginosa* (PA), *S*. *aureus* (SA), and *C*. *albicans* (CA) strains in mono-species biofilm treated with DNase I (B), pASP (C) and Pf1 bacteriophage (D) at concentrations of 5 μg/mL, 10 μg/mL and 0.1 mg/mL, respectively and co-administrated with CSA-13 at dose of 20 μg/mL (E-G). Recorded values were compared to the anti-biofilm activity of CSA-13 alone (A). Green bars indicate percentage of bacteria grown. Results are presented as mean ± SD from 5 strains with three repetitions. * indicates statistical significance when compared to Pf-negative isolates.
(TIF)

S4 Fig Rheological measurements of mono-species biofilm formed by *P*. *aeruginosa* (PA), *S*. *aureus* (SA), and *C*. *albicans* (CA) strains. Shear storage modulus as a function of shear strain amplitude (A), phase shift as a function of shear strain amplitude (B), storage modulus as a function of oscillation frequency (C), and dynamic viscosity (D) were measured.
(TIF)

S5 Fig Rheological measurements of polymicrobial biofilms formed by Pf-negative *and* Pf-positive *P*. *aeruginosa* isolates with *S*. *aureus*, and *C*. *albicans* strains in dual-species mixtures upon treatment with single agents. Shear storage modulus as a function of shear strain amplitude (A-E) and dynamic viscosity (F-J). Rheological properties were made for polymicrobial biofilms formed by Pf-negative *and* Pf-positive *P*. *aeruginosa* isolates with *S*. *aureus*, and *C*. *albicans* strains in dual-species mixtures. Biofilm was measured in the presence of CSA-13 (B, G), DNase I (C, H) pASP (D, I) and Pf1 bacteriophage (E, J) at concentrations of 20 μg/mL, 5 μg/mL, 10 μg/mL and 0.1 mg/mL, respectively. Recorded values were compared to the control—without treatment (A, F).
(TIF)

S6 Fig Rheological measurements of polymicrobial biofilms formed by Pf-negative *and* Pf-positive *P*. *aeruginosa* isolates with *S*. *aureus*, and *C*. *albicans* strains in dual-species mixtures upon treatment with co-administrated agents. Shear storage modulus as a function of shear strain amplitude (A-E) and dynamic viscosity (F-J). Rheological properties were made for polymicrobial biofilms formed by Pf-negative *and* Pf-positive *P*. *aeruginosa* isolates with *S*. *aureus*, and *C*. *albicans* strains in dual-species mixtures. Biofilm was measured with CSA-13 at dose of 20 μg/mL in the co-administrated with DNase I (A, D) pASP (B, E) and Pf1 bacteriophage (C, F) at concentrations of 5 μg/mL, 10 μg/mL and 0.1 mg/mL, respectively.
(TIF)

S1 File (ZIP)
