# Supplementary figures and images for: Ceragenin-mediated disruption of Pseudomonas aeruginosa biofilms
Source: PLoS One. 2024 Feb 12;19(2):e0298112. doi: 10.1371/journal.pone.0298112 (PMC10861078; doi:10.1371/journal.pone.0298112)

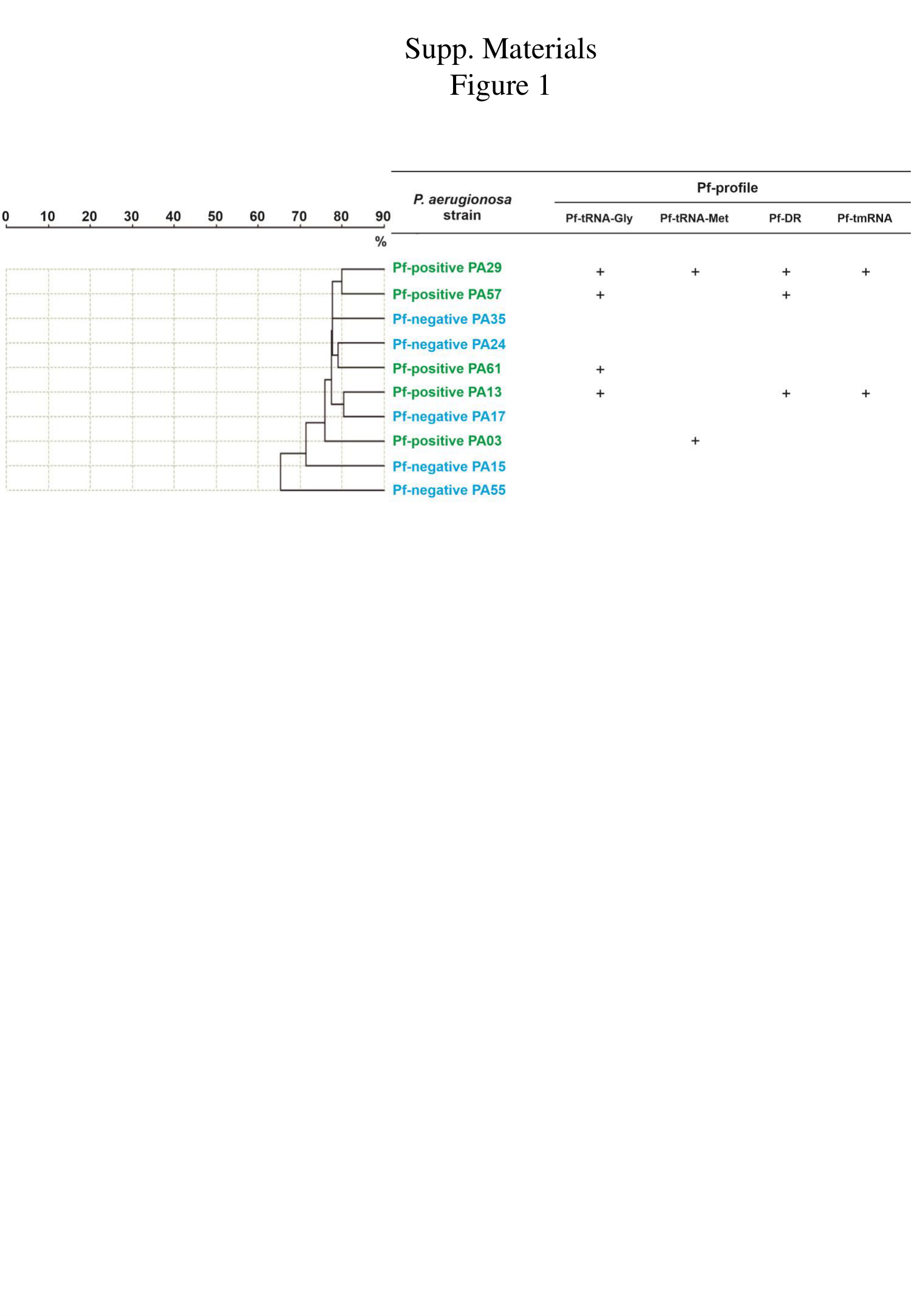

Supplement: S1 Fig — Analysis was performed using ‘taxonomy module’ in Saramis v4.12 Vitek MS-Plus RUO software. (TIF) [file pone.0298112.s001.tif]

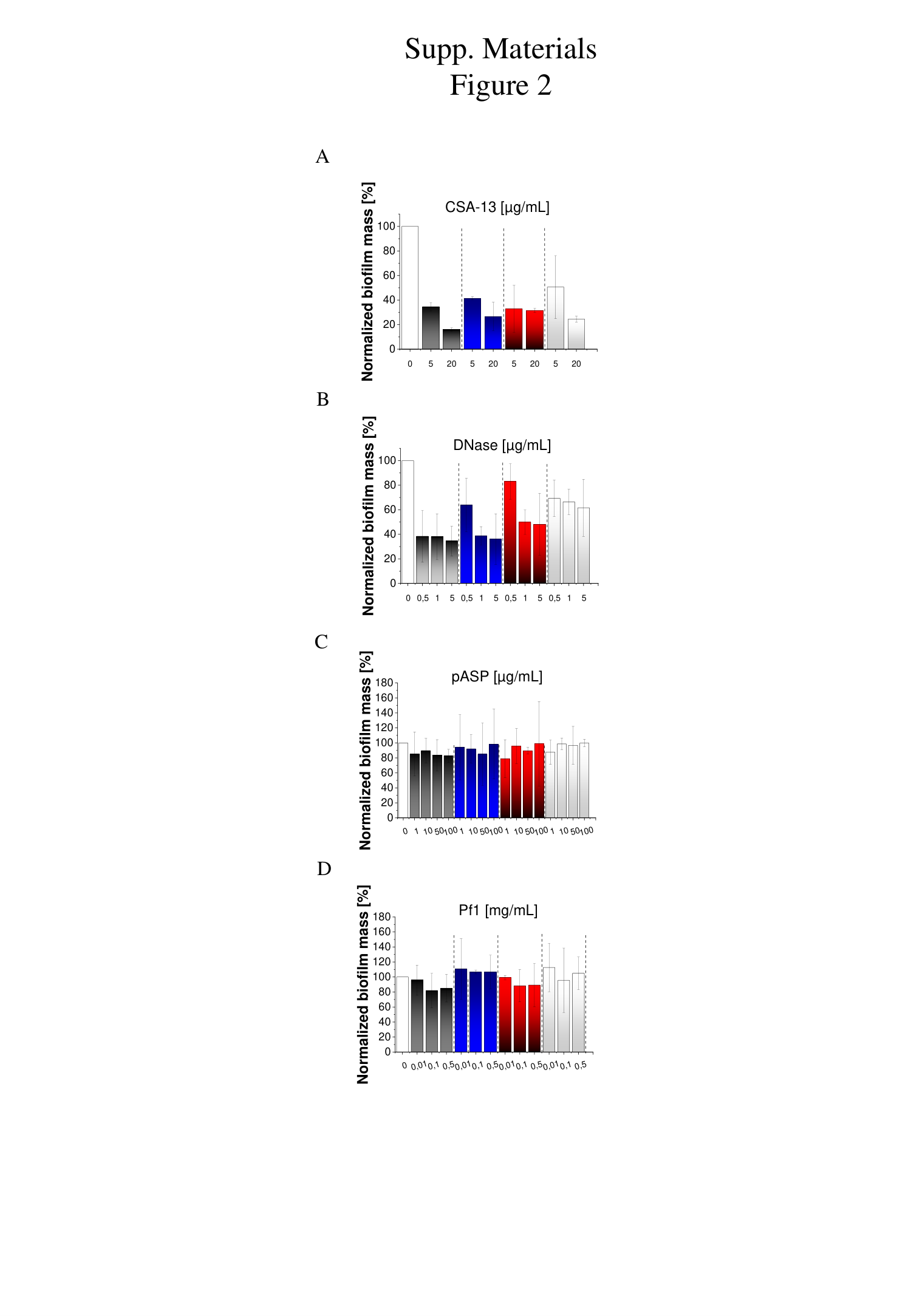

Supplement: S2 Fig — Relative mass of polymicrobial biofilms formed by Pf-negative and Pf-positive P. aeruginosa isolates (black and blue bars, respectively), S. aureus (SA, red bars), and C. albicans (CA, grey bars) strains in dual-species mixtures treated with CSA-13 (A), DNase I (B), pASP (C) and Pf1 bacteriophage (D) at concentrations ranging from 5 to 20 μg/mL, 0.05 to 5 μg/mL, 1 to 100 μg/mL and 0.01 to 0.5 mg/mL, respectively. Results are presented as mean ± SD from 5 strains with three repetitions. * indicates statistical significance when compared to Pf-negative P. aeruginosa isolates. (TIF) [file pone.0298112.s002.tif]

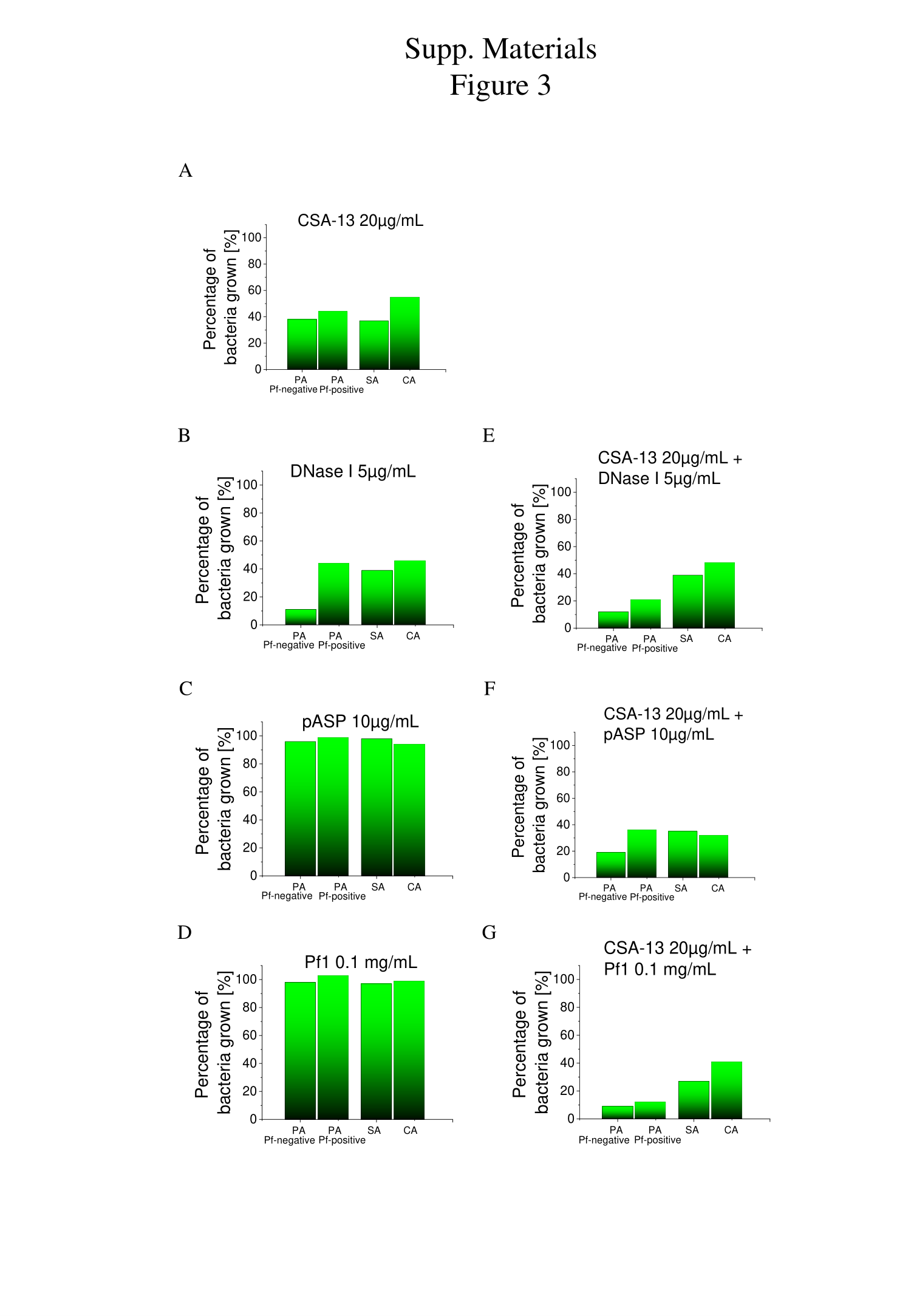

Supplement: S3 Fig — Viability of biofilms formed by P. aeruginosa (PA), S. aureus (SA), and C. albicans (CA) strains in mono-species biofilm treated with DNase I (B), pASP (C) and Pf1 bacteriophage (D) at concentrations of 5 μg/mL, 10 μg/mL and 0.1 mg/mL, respectively and co-administrated with CSA-13 at dose of 20 μg/mL (E-G). Recorded values were compared to the anti-biofilm activity of CSA-13 alone (A). Green bars indicate percentage of bacteria grown. Results are presented as mean ± SD from 5 strains with three repetitions. * indicates statistical significance when compared to Pf-negative isolates. (TIF) [file pone.0298112.s003.tif]

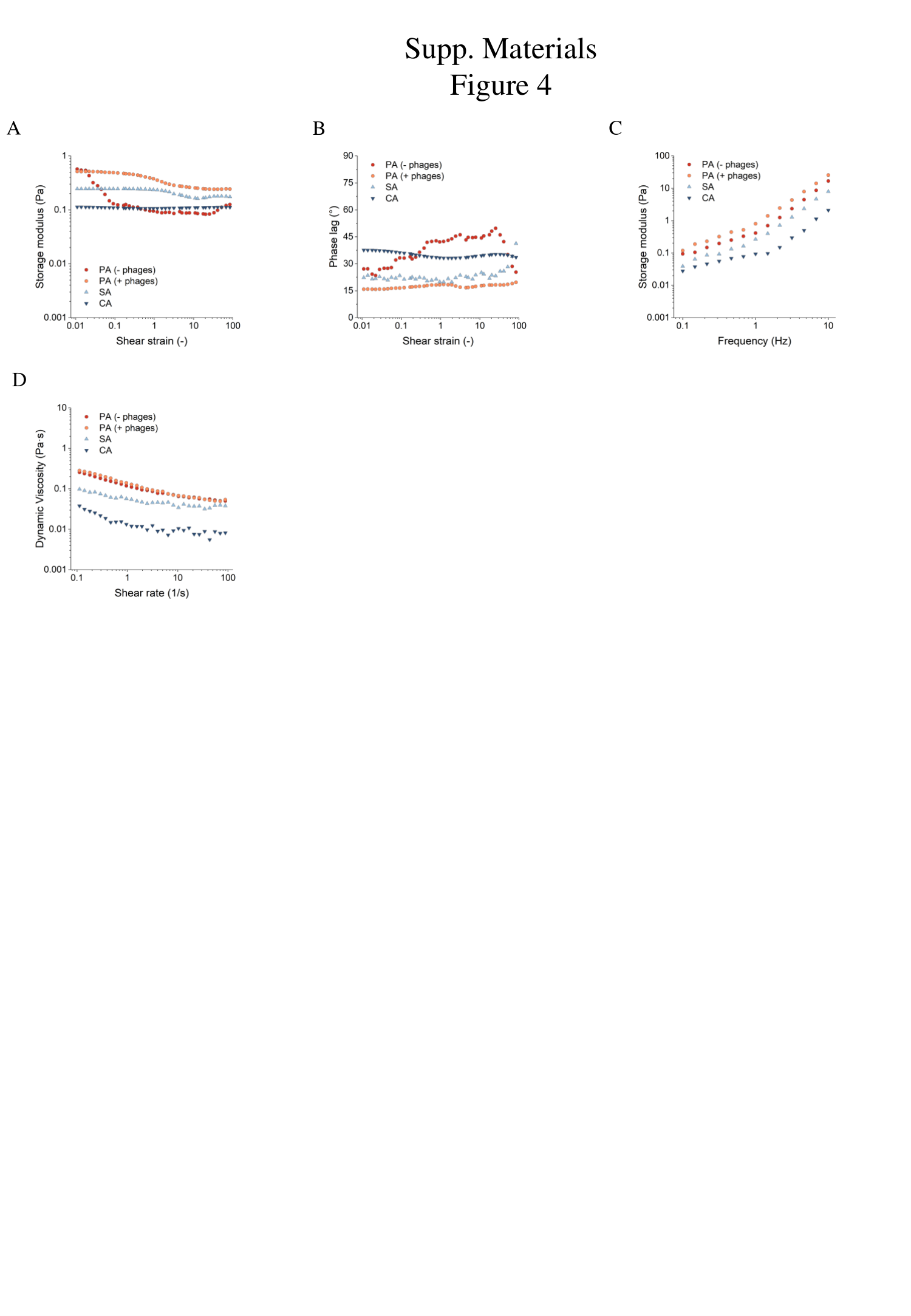

Supplement: S4 Fig — Shear storage modulus as a function of shear strain amplitude (A), phase shift as a function of shear strain amplitude (B), storage modulus as a function of oscillation frequency (C), and dynamic viscosity (D) were measured. (TIF) [file pone.0298112.s004.tif]

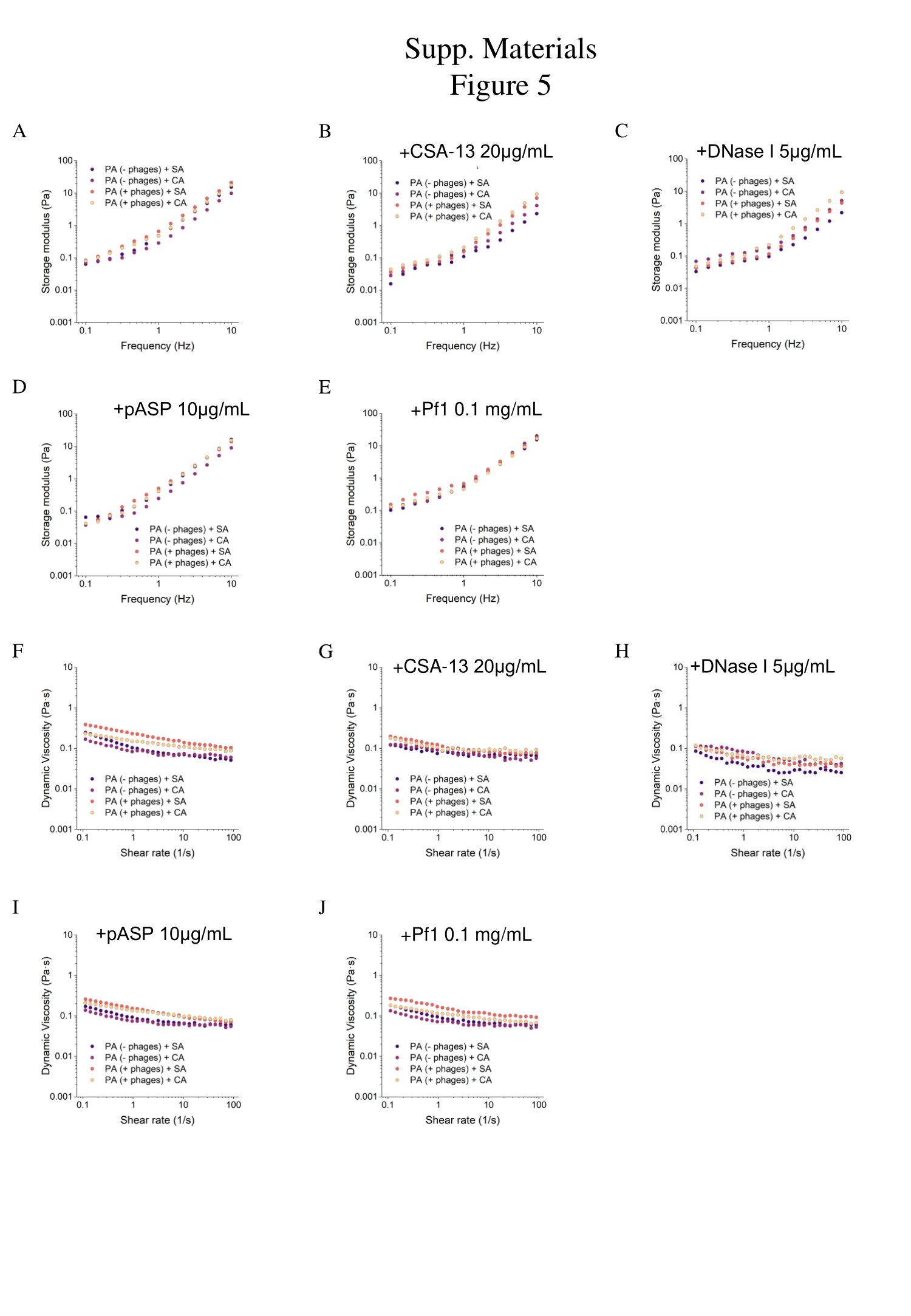

Supplement: S5 Fig — Shear storage modulus as a function of shear strain amplitude (A-E) and dynamic viscosity (F-J). Rheological properties were made for polymicrobial biofilms formed by Pf-negative and Pf-positive P. aeruginosa isolates with S. aureus, and C. albicans strains in dual-species mixtures. Biofilm was measured in the presence of CSA-13 (B, G), DNase I (C, H) pASP (D, I) and Pf1 bacteriophage (E, J) at concentrations of 20 μg/mL, 5 μg/mL, 10 μg/mL and 0.1 mg/mL, respectively. Recorded values were compared to the control—without treatment (A, F). (TIF) [file pone.0298112.s005.tif]

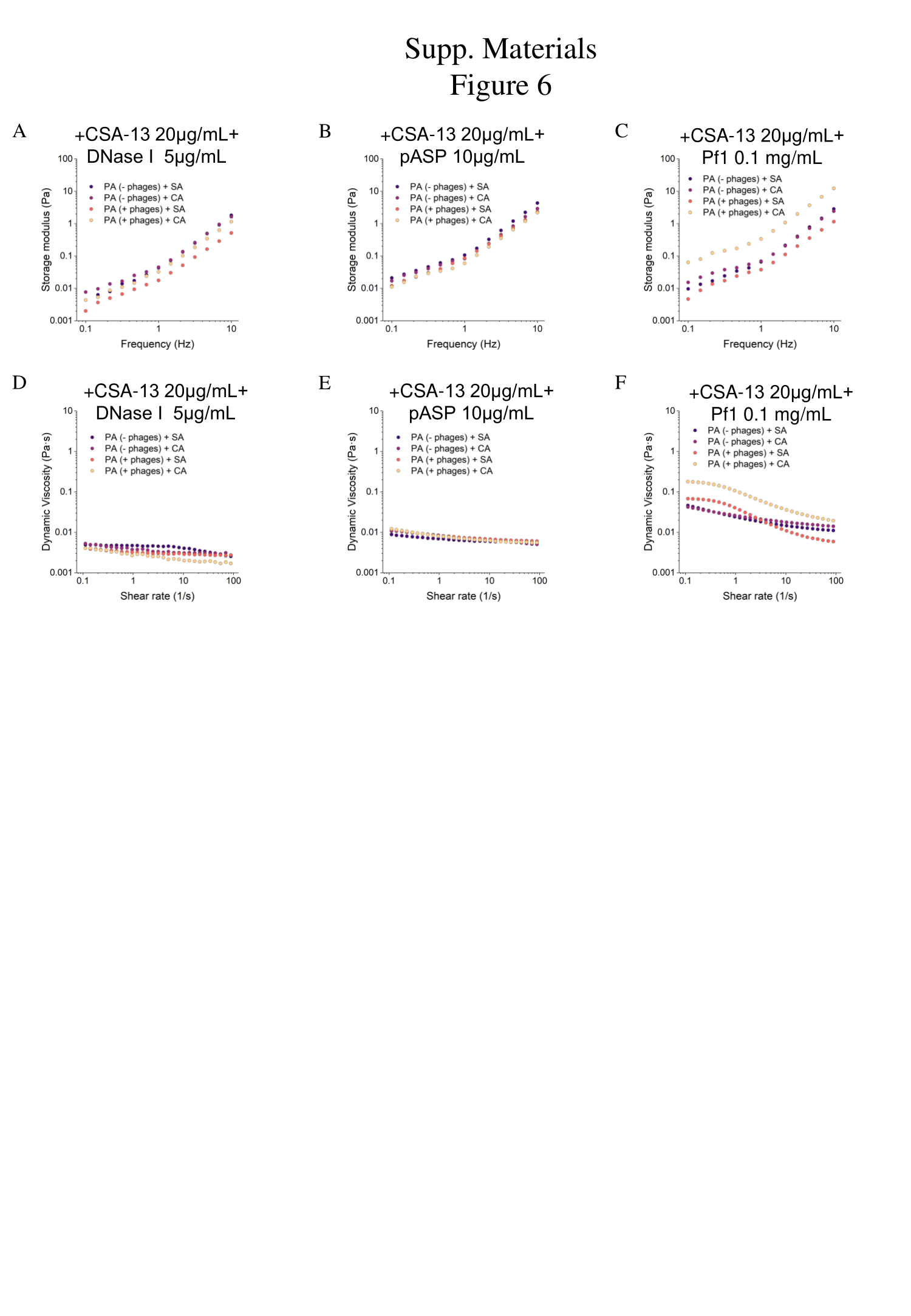

Supplement: S6 Fig — Shear storage modulus as a function of shear strain amplitude (A-E) and dynamic viscosity (F-J). Rheological properties were made for polymicrobial biofilms formed by Pf-negative and Pf-positive P. aeruginosa isolates with S. aureus, and C. albicans strains in dual-species mixtures. Biofilm was measured with CSA-13 at dose of 20 μg/mL in the co-administrated with DNase I (A, D) pASP (B, E) and Pf1 bacteriophage (C, F) at concentrations of 5 μg/mL, 10 μg/mL and 0.1 mg/mL, respectively. (TIF) [file pone.0298112.s006.tif]
